# Congenital Hyperinsulinism India Association: An Approach to Address the Challenges and Opportunities of a Rare Disease

**DOI:** 10.3390/medsci13020037

**Published:** 2025-04-01

**Authors:** Jaikumar B. Contractor, Venkatesan Radha, Krati Shah, Praveen Singh, Sunil Tadepalli, Somashekhar Nimbalkar, Viswanathan Mohan, Pratik Shah

**Affiliations:** 1 GMERS Medical College, Panchmahal, Godhra 389120, Gujarat, India; jaicontractor02@gmail.com; 2 Congenital Hyperinsulinism India Association (CHIA), Anand 388325, Gujarat, India; sunil.tadepalli@labnetworx.com (S.T.); somu_somu@yahoo.com (S.N.); 3 Madras Diabetes Research Foundation (ICMR Collaborating Centre of Excellence), Chennai 600086, Tamil Nadu, India; radharv@yahoo.co.in (V.R.); drmohans@diabetes.ind.in (V.M.); 4 One Centre for Genetics, Vadodara 390007, Gujarat, India; drkratishah@gmail.com; 5 Pramukhswami Medical College, Bhaikaka University, Karamsad 388325, Gujarat, India; praveenrs@charutarhealth.org; 6 Labnetworx Health IT LLP, New Delhi 110092, India; 7 The Royal London Childrens Hospital, Barts Health NHS Trust, E1 1FR & Queen Mary University of London, London EC1M 6BQ, UK

**Keywords:** glucose metabolism disorders, healthcare professional consortium, parent support groups, patient information leaflets, database registries, patient advocacy, genetic diseases-inborn

## Abstract

India’s population complexity presents varied challenges in genetic research, and while facilities have gained traction in tier-1 and -2 cities, reliance on international collaborations often delays such investigations. COVID-19 further exacerbated the issues with such sample sharing. Congenital Hyperinsulinism (CHI) is a rare genetic disorder of pancreatic β-cells causing hypoglycaemia in children due to abnormal insulin secretion. Given India’s high birth rate and consanguineous populations, annual CHI cases are estimated to be around up to 10,000, with up to 50% having unexplained genetic causes. Diffuse or atypical lesions in such patients often necessitate near-total-pancreatectomy, risking pancreatic exocrine insufficiency and diabetes, requiring lifelong therapy. Also, novel genetic variations complicate accurate diagnosis, risk assessment, and counselling, emphasising the need for rapid genetic assessment to prevent neurological injuries and inform treatment decisions. Despite significant efforts at many institutes, there are no dedicated organisations for CHI in India. With the implementation of the National Policy for Rare Diseases 2021, we plan to form a non-profit organisation, “Congenital Hyperinsulinism India Association (CHIA)”, comprising paediatric endocrinologists, paediatricians, geneticists, and independent researchers. The aims of this association are to generate a national database registry of patients, formulate a parent support group and CHIA consortium, design patient information leaflets, as well as foster genomic collaborations and promote clinical trials. Such steps will help sensitise the health authorities and policy makers, urging them to improve the allocation of health budgets for rare diseases, as well as empower patients and their families, contributing towards a better quality of life.

## 1. Introduction

### 1.1. Scenario of Genetic Diseases in India

India holds the distinction of being the most populous country in the world with 1.44 billion people, representing 17.5% of the global population. The population is also expected to increase at an annual rate of 1.0 percent from 2011 (1.21 billion) to 2036 (1.51 billion), currently standing at 1.39 billion [1]. The country is believed to have been inhabited by migrants from the African subcontinent approximately 65,000 years ago, followed by numerous events of migration and invasion causing a population admixture that has led to highly heterogeneous population groups [2,3,4,5]. At present, there are over 4000 anthropologically distinct groups and 21 different languages with various dialects in India. Recently, the Indian Genome variation consortium identified polymorphism in 900 genes from 55 different population groups of India that are now catalogued in the Indian Genome Variation Browser [6]. With such vast geographical and multi-linguistic population in a socially and culturally deep-rooted religious/caste system, the genetic make-up of the population is further complicated by the high degree of endogamy, with consanguineous marriages as common as 20–30% in specific population groups [7,8]. This places India in a unique position, with the possibility of clusters of specific diseases and founder mutations [9].

The National Health Policy, 2017 of India envisaged an increase in expenditure of 2.5% of the GDP (Gross Domestic Product) by 2025, while the central and state government’s budgeted expenditure on the health sector reached 2.1% in 2021–22 against 1.3% in 2019–20 [10]. Currently, India spends 3.73% of its GDP on its health budget [11], focusing on services providing free and subsidised public health services as executed by all 49 state governments, provided by the Government of India under the Ministry of Health and Family Welfare (https://pib.gov.in/PressNoteDetails.aspx?NoteId=153237&ModuleId=3&reg=3&lang=1 accessed on 24 October 2024). These are accessed by 25–30% of the overall population, with the rest of the population being served by heterogeneous private health services managed by various corporate organisations and individual practices. Here, geographic and economic disparity plays a pivotal role in the availability of infrastructural resources between the urban and rural population, as well as the economically privileged and deprived classes [12,13].

Such factors pose a considerable challenge for genetic studies and genomic medicine research in India. However, the growing significance of molecular genetics and cytogenetics in diagnosis, as well as the management of many disorders, have led to state-of-the-art molecular diagnostic and counselling facilities in India. Most of the genetic services in terms of clinical as well as molecular geneticists, genomic laboratories and counselling infrastructure are available at tier-1 and -2 cities and premier institutes, while their availability across tier-3 and -4 cities, as well as rural areas, is much lower. Almost all genetic disorders have been reported in India. While such research often comes from a few institutions and individuals, there is a need to establish nationwide prevalence and create a registry of rare genetic disorders. This would also enable the genetic evaluation of a majority of the population and develop a national public health programme for carrier or newborn screening for genetic disorders, as well as advance research in understanding the pathogenesis and discovering newer treatment modalities for genetic disorders and in areas like gene mapping and gene and stem cell therapy [14,15,16].

In the past, genetic investigations have primarily been conducted outside the country on a peer-to-peer basis, with individual investigators coordinating sample collection, storage, maintenance, and redistribution. This has resulted in a challenging turn-around time, often consuming the critical period of a serious illness. And as the community demands have risen, such commitments have become increasingly burdensome, costly, and legally and ethically complex. Recently, COVID-19 laid bare the challenges of sharing clinical and other biological samples. Following the pandemic, there is a demand for the wider adoption of electronic legal agreements for sample transfer and increased harmonisation, interoperability, and searching capacity for sample collection. These issues are unlikely to go away, unless there is more support and stronger incentives from funders and publishers for infrastructure; meticulous and organised utilisation of the available resources and current efforts, which work in silos; and their unification into a national cause, particularly in the context of rare genetic disorders [17].

### 1.2. Congenital Hyperinsulinism

Congenital Hyperinsulinism (CHI) is a rare genetic disorder of the pancreatic β-cells known to cause recurring episodes of hypoglycaemia due to dysregulated insulin secretion, leading to dangerously low blood glucose concentrations in infants and children [18]. Such inappropriate insulin secretion can also suppress the production of ketone bodies, which serve as an alternative fuel for the brain during hypoglycaemia. This lack of glucose and the deprivation of alternative fuels increase the risk of brain damage in these patients, causing an increase in long-term morbidity as well as mortality [19,20]. With an annual birth rate of 25 million children, India accounts for nearly one-fifth of the world’s annual child births. Up to 1.7 million newborn babies are estimated to be born with birth defects/genetic conditions, with a report supporting 9% of newborns have birth defects [21]. This accounts for at least 625 children estimated to be born with CHI annually, at an incidence of 1 in 40,000 live births in the general population, and this could go up to 10,000 per year, with an incidence of 1 in 2500 in populations with high rates of consanguinity [22,23]. The associated genetic loci causing monogenic forms have been identified as *ABCC8* and *KCNJ11* [24,25,26,27,28,29], *GLUD1* [30,31], *GCK* [32], *HADH1* [33], *SLC16A1* [34,35], *INSR* [36,37], *UCP2* [38,39], *HNF4α* and *HNF1α* [40,41,42,43,44,45], and *HK1* [46]. More than over 20 syndromic forms of CHI have reported so far with multi-systemic involvement, the commonly reported ones being *KCNQ1*/*CDKN1C* (Beckwith–Wiedemann syndrome) and *KMT2D/KDM6A* (Kabuki Syndrome), amongst other syndromic forms [47,48,49,50,51,52,53]. However, despite significant advances which have expanded our understanding of the pathophysiology of this disorder, the cause of CHI remains unknown in up to 50% of, patients suggesting the existence of additional genetic loci that have yet to be discovered [54]. Moreover, up to one-third to one-half of K_ATP_ channel mutations detected by genetic testing are novel variants that are interpreted as variants of unknown significance [55]. The optimal management of hyperinsulinism is dependent upon the underlying cause, and it highlights the importance of a good turn-around time of the genetic evaluation, which is also essential to prevent neuroglycopenic brain damage. Also, the characterisation of novel variants and their associated phenotypes is essential for the accurate diagnosis of affected children, improving the interpretation of genetic tests, counselling families about recurrence risks, and identifying other family members at risk of hypoglycaemia [56].

**Figure 1:**
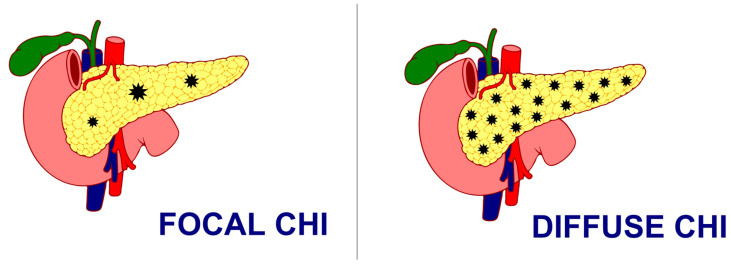
Focal CHI and Diffuse CHI.

Histologically, CHI has been classified as either a focal form with the involvement of a small area of islet cell expansion and minimal exocrine tissue involvement or a diffuse form involving the entire pancreas and nucleomegaly of some islet cells or atypical forms with a combination of both focal and diffuse lesions [57] (Figure 1). While the management of CHI includes nutritional, medical, and surgical intervention depending on the underlying histologic and genetic subtype, focal lesionectomy is the preferred curative treatment for focal CHI when a complete resection is achieved. But the management of diffuse lesions, which account for the majority of the cases, still poses a significant challenge with near-total-pancreatectomy, i.e., resection of 95–98% of pancreatic tissue as a last resort in preventing hypoglycaemic brain damage, especially in severe cases where sufficient glycaemic control cannot be achieved, even though the combination of different medications and nutritional supplements poses a persistent risk of hypoglycaemia and subsequent neurodevelopmental delay [58,59,60]. However, even surgical interventions with near-total-pancreatectomy have been proven to be not curative in such patients, with often inconsistent and unsatisfactory outcomes, ranging from persisting hypoglycaemia (up to 60%), hyperglycaemia (almost 100% at 11 years post-surgery), and exocrine pancreatic insufficiency (almost 50%) [61]. Moreover, almost 96% of the patients reported the incidence of insulin-dependent diabetes mellitus 10–15 years after near-total-pancreatectomy, 77% after 7 years, and 13% soon after surgery [57].

It is very important to follow-up children with this condition in view of some genetic forms that can lead to diabetes later in life. Also, children with pancreatectomy are at a higher risk of developing pancreatic exocrine insufficiency and diabetes later in life.

### 1.3. Indian Organisations for CHI

With significant advances in gene mapping achieved through molecular genetics and exome sequencing, there exists substantial research on CHI in India, especially from leading institutes like the All India Institute of Medical Sciences, New Delhi; Postgraduate Institute of Medical Education and Research, Chandigarh; Madras Diabetic Research Foundation, Chennai; amongst many others. There has also been a significant amount of work on CHI across the country that includes prominent clinicians in government and private institutions. However, while there are various organisations in India working in the field of genetic disorders, as well as rare diseases [62], our literature review did not find any particular organisation working for CHI in India. With the implementation of National Policy for Rare Disease (NPRD), 2021 by the Ministry of Health and Family Welfare, the Government of India aims to lower the incidence and prevalence of rare diseases based on an integrated and comprehensive preventive strategy [63]. Thus, we plan to form the Congenital Hyperinsulinism India Association (CHIA).

### 1.4. Global Organisations for CHI

○ Congenital Hyperinsulinism International (https://congenitalhi.org)–Congenital Hyperinsulinism International is a grass-root level organisation founded in 2005 by the concerned parents of children with hyperinsulinism. It is a global organisation supported in its work by a scientific advisory team comprising eleven leading hyperinsulinism world specialists. It remains dedicated to increasing awareness; providing education, information, and support to those living with the disease; advocating on behalf of the patients for better treatments and access to care; and improving the lives of the babies, children, and adults affected by the disorder. It also supports medical research for improved therapies, potential cures, and timely diagnosis.
○ Congenital Hyperinsulinism Charity UK (CHC-UK).
○ There are also family support groups reported in Germany, Spain, and various other countries.

**Congenital Hyperinsulinism India Association (CHIA) https://www.hyperinsulinism-india.org/ (accessed on 10 November 2024)**

Considering the above lacunae and to address the challenges in the management of such disorders, we have established the non-profit organisation, “Congenital Hyperinsulinism India Association”—CHI India Association (CHIA)—to dedicate our efforts in the field of Congenital Hyperinsulinism.

**Congenital Hyperinsulinism India (CHIA)—Vision**

○ To raise awareness of Congenital Hyperinsulinism as a rare genetic disorder among the public and healthcare professionals across India and contribute towards a better quality of life for the children and their families.

**Congenital Hyperinsulinism India Association (CHIA)—Mission**

At CHIA, we dedicate our efforts in creating a one-stop solution for CHI in India. We plan to collaborate with individual clinicians as well as researchers and other organisations to unify efforts in the following:

○ Offer prompt genetic testing for clinically and biochemically confirmed cases of CHI at the CAP (College of American Pathologists)- and NABL (National Accreditation Board for Testing and Calibration Laboratories)-accredited genetic centre in India to provide quick turn-around of results.
○ Generate a national database and registry of patients for collaborative research, improving access to diagnostics and treatment.
○ Formulate a parent support group and CHI-India Association consortium to raise awareness amongst families (with different vernacular regional languages) and healthcare professionals through national conferences and seminars, as well as government supported programmes.
○ Design patient information leaflets (in different vernacular regional languages) to provide uniform and adequate care as well as ease of access to information.
○ Identify and support potential centres of excellence in the country to expedite the diagnostic services in terms of genetic evaluation and imaging, as well as treatment modalities.
○ Foster genomic collaborations with international organisations, as well as genetic centres (US and UK), to promote research collaborations in identifying and characterising novel functional gene mutations.
○ Promote clinical trials for newer investigations and treatment modalities so children and families in India can obtain access to newer therapies.

## 2. Discussion

### 2.1. CHIA Patient Registry

An organised system to collect uniform data and evaluate specified outcomes in patients and families with CHI has been designed in accordance with the AHRQ guidelines [64]. The use of national registries for quality improvement purposes in rare diseases has been advocated in many countries and commonly serves the purpose of generating and formalising a data source, performing natural history studies, evaluating the association between clinical characteristics and health outcomes, understanding the phenotype–genotype correlations, establishing a network for future diagnostic and treatment studies, as well as supporting the patients to offer a more holistic care [65,66]. The CHIA registry consists of a series of questions, including demographic details, clinical history biochemical profile and treatment details, amongst others, to be filled by the patients/parents/guardians/consulting clinicians. The registry positions the patients and their families in a unique position to provide information related to their symptoms, functional status, quality of life and the overall burden of the disease to generate real-world data, and such patient reported outcomes could play an integral role in providing additional insight into the medical and day-today experience of living with a rare disease, as well as in clinical trial designs and regulatory decision making [67,68,69]. A GDPR (General Data Protection Regulations)-compliant database will also enable the electronic access of healthcare records in a mutually exclusive, ethical, and patient-friendly manner that could open more avenues for research in potential new treatments and improve quality of life.

### 2.2. CHIA—Healthcare Professionals’ Consortium

A CHI-India Association consortium has been developed that brings together a cadre of healthcare professionals like researchers, geneticists, clinicians, collaborators, as well as parents/patients to share relevant skills, experience and expertise in the field of CHI and thereby create a more user-friendly systems of care. The formally structured CHIA consortium is devised on the structural approach depending on the unique circumstances and problems pertaining to CHI in India. The consortium comprising its executive board functions to advance medical research through awareness campaigns outreach programmes and conferences along with community-wide needs assessment planning, enabling uniform high-quality data collection and analysis, data sharing, developing collateral resources and facilitating collaboration and information sharing across the network. More importantly, the consortium is envisaged to initiate cross-cutting collaborations between various national organisations of repute like Indian Academy of Pediatrics (IAP), National Neonatology Forum India (NNF India), ISPAE (Indian Society for Pediatric and Adolescent Endocrinology) as well as collaborate with global cooperation platforms like CHI International. This will enable us to develop national guidelines as well as identify centres of excellence for standardised CHI care in the country that will enable to create opportunities for advancing diagnostic and treatment options, including clinical trials for improved healthcare [70].

### 2.3. CHIA—Parent Support Group

A CHIA parent support group has been established to create a collective effort for awareness, policy issues, advocacy, research, and medical advancements, as well as emotional and psychological support to all patients and families with CHI. Similar parent support groups have yielded encouraging results, feeling empowered and a sense of belonging as they have been able to connect with each other and the healthcare professionals to provide emotional and financial support, as well as skills to deal with the day-to-day issues of raising children with rare disabilities [71,72].

### 2.4. CHIA—Information Leaflets

Considering the linguistic disparity and socio-cultural differences, information leaflets have been designed for patients as well as healthcare workers to create a high-quality source of good, accessible, and comprehensive information on the etio-pathogenesis, role of involved genes, and treatment options for CHI amongst others. With the advent of information and technology, patients have become increasingly engaged in their own healthcare, and these information leaflets serve in effectively communicating the information as well as in educating the stakeholders about the disease. It also provides a scope for creating a mutually beneficial partnership between patients, their families, and healthcare services that respects individual needs and values as well as demonstrates how shared decision making and patient empowerment enhance patient adherence to treatment [73,74,75]. Information leaflets that cater to the readability of individual patients, meet a global standard, and set up standardised guidelines to bring CHI on a uniform platform for all stakeholders have been designed (regional vernacular languages are in process). The way forward is to design and translate the leaflets for individual regional vernacular languages; disseminate these leaflets through awareness campaigns, social media platforms, and practice patient participation groups; and establish policies and protocols for patient care and management [76,77].

### 2.5. CHIA—Genetic Analysis and Genomic Collaborations

We have established communication channels with consultant paediatric endocrinologists and neonatologists along with clinical and molecular geneticists across India to coordinate investigations on next generation sequencing for the children and sanger sequencing for the parents. Information sheets about the CHI India Association, consent form, and a clinical proforma for generating a database are shared with these collaborators to conduct prompt testing and free-of-charge genetic analysis at the CAP (College of American Pathologists)- and NABL (National Accreditation Board for Testing and Calibration Laboratories)-accredited genetic centre “MDRF—Madras Diabetic Research Foundation” in India (www.mdrf.in & www.monogenicdiabetes.in (accessed on 10 November 2024)) to reduce the turn-around time of results. Moreover, with over one-third to one-half of the cases estimated to be of novel variants with unknown significance, we have collaborated with Genomic Medicine, University of Exeter Medical School and the Centre for Endocrinology, William Harvey Research Institute, Queen Mary University of London to identify and characterise the genes with novel mutations, as well as undertake functional work in future multi-centric genetic studies.

### 2.6. CHIA–Centres of Excellence

We aim to identify and collaborate with potential centres of excellence in the country to expedite the diagnostic services in terms of genetic evaluation and imaging, as well as treatment modalities, of rare diseases under the National Policy for Rare Diseases, 2021 [63].

## 3. Conclusions

Raising awareness regarding the burden of Congenital Hyperinsulinism in India to the lay population and medical practitioners is the need of the hour. Steps like CHIA may help to sensitise the health authorities and policy makers towards the need for an improvement in the allocation of health budgets, especially towards a rare disorder such as this. A national database for this rare disorder will be crucial in the empowerment of patient advocacy groups and proactive involvement of policy makers in the implementation of such programmes. While we understand that achieving such goals dedicated to CHI in India is hindered by several challenges, including the lack of awareness among the public and healthcare professionals, socio-economic disparities, competing healthcare priorities, and high treatment costs coupled with research and infrastructure gaps. At CHIA, we strongly urge the Government of India—including DST, ICMR, DBT, CSIR, and other agencies—along with philanthropists, corporate houses, pharmaceutical companies, private genetic laboratories, and foundations both in India and abroad, to join us in the fight against CHI in India and provide crucial support for such initiatives. We also aim to collaborate with national/international paediatric as well as neonatology organisations for furthering our goals. We offer memberships for the CHI-India Association consortium and CHI-India Association parent support group, and the links and registration page can be found on our website. Further information about the CHI-India Association can be found on www.hyperinsulinism-india.org.

## Data Availability

The original data presented in this study are openly available at www.hyperinsulinism-india.org (accessed on 5 January 2023).

## Abbreviations

The following abbreviations are used in this manuscript:

AHRQ	Agency for Healthcare Research and Quality
CAP	College of American Pathologists
CHI	Congenital Hyperinsulinism
CHIA	Congenital Hyperinsulinism India Association
CSIR	Council of Scientific and Industrial Research
DBT	Department of Biotechnology
DST	Department of Science and Technology
GDP	Gross Domestic Product
GDPR	General Data Protection Regulations
IAP	Indian Association of Pediatrics
ICMR	Indian Council of Medical Research
ISPAE	Indian Society of Pediatric and Adolescent Endocrinologists
MDRF	Madras Diabetes Research Foundation
NABL	National Accreditation Board for Testing and Calibration Laboratories
NNF	National Neonatology Forum
NPRD	National Policy for Rare Diseases
